# A pilot, randomized, double‐blind, placebo‐controlled trial to assess the safety and efficacy of a novel Boswellia serrata extract in the management of osteoarthritis of the knee

**DOI:** 10.1002/ptr.6338

**Published:** 2019-03-06

**Authors:** Muhammed Majeed, Shaheen Majeed, Narayanan K. Narayanan, Kalyanam Nagabhushanam

**Affiliations:** ^1^ Sami Labs Limited Research & Development Division Bangalore India; ^2^ Sabinsa Corporation Research & Development Division Payson Utah; ^3^ Sabinsa Corporation Research & Development Division East Windsor New Jersey

**Keywords:** anti‐inflammatory, Boswellia serrata extract, cathepsin G, high‐sensitive C‐reactive protein, knee osteoarthritis, microsomal prostaglandin E synthase‐1

## Abstract

A double‐blind, placebo‐controlled human trial was conducted to evaluate the safety and efficacy of a standardized oral supplementation of *Boswellin*®, a novel extract of Boswellia serrata extract (BSE) containing 3‐acetyl‐11‐keto‐β‐boswellic acid (AKBBA) with β‐boswellic acid (BBA). A total of 48 patients with osteoarthritis (OA) of the knee were randomized and allocated to the BSE and placebo groups for intervention. Patients were administered BSE or placebo for a period of 120 days. The trial results revealed that BSE treatment significantly improved the physical function of the patients by reducing pain and stiffness compared with placebo. Radiographic assessments showed improved knee joint gap and reduced osteophytes (spur) confirming the efficacy of BSE treatment. BSE also significantly reduced the serum levels of high‐sensitive C‐reactive protein, a potential inflammatory marker associated with OA of the knee. No serious adverse events were reported. This is the first study with BSE conducted for a period of 120 days, longer than any other previous clinical trial on patients with OA of the knee. The findings provide evidence that biologically active constituents of BSE, namely, AKBBA and BBA, act synergistically to exert anti‐inflammatory/anti‐arthritic activity showing improvement in physical and functional ability and reducing the pain and stiffness.

**Abbreviations**
5‐LOX: 5‐lipoxygenase
ABBA: 3‐acetyl‐β‐boswellic acid
AKBBA: 3‐acetyl‐11‐keto‐β‐boswellic acid
BA: Boswellic acid
BBA: β‐boswellic acid
BSE: Boswellia serrata extract
European Q5D: European Quality of life‐5 Dimension
hs‐CRP: high‐sensitive C‐reactive protein
KBBA: 11‐keto‐β‐boswellic acid
LPS: lipopolysaccharide
NSAID: nonsteroidal anti‐inflammatory drug
OA: osteoarthritis
VAS: visual analog scale
WOMAC: Western Ontario McMaster Index

## 1 INTRODUCTION

Osteoarthritis (OA) is an age‐related, disabling degenerative joint disorder worldwide, often associated with chronic unresolved inflammation, characterized by joint pain, decreased mobility, and negative impact on quality of life (QOL; Glyn‐Jones et al., 2015). OA of the knee is the most common form of arthritis and has a high prevalence rate compared with other types of OA. Overall, an estimated 43.5% American adults were affected by arthritis in 2013–2015, reflecting a net increase of about 1 million people per year (Barbour, Helmick, Boring, & Brady, 2017). By 2040, that number is expected to raise to nearly 78.4 million of the projected total adult population (Barbour et al., 2017). One of the primary focus of OA medication is to reduce pain and the use of acetaminophen, and nonsteroidal anti‐inflammatory drugs (NSAIDs) are currently the mainstay of pharmacotherapy for OA of the knee. Unfortunately, many patients do not respond to the above treatments. NSAIDs are known to be associated with gastrointestinal, renal, and cardiovascular risks (Umar et al., 2014). Therefore, search for herbal agents with anti‐inflammatory properties without adverse side effects for the treatment of OA is on the rise.

Interestingly, the gum resin extracted from a well‐known herb Boswellia serrata Roxb. ex Colebr. (family: Burseraceae), also called Indian frankincense or Salai guggal, has been used in traditional Ayurvedic medicine in India for centuries as a remedy for the treatment of chronic inflammatory diseases, including OA (Ammon, 2016; Ernst, 2008). In the recent past, the potent anti‐inflammatory, anti‐arthritic, and analgesic activities of the gum resin extract from B. serrata has gained much attention (Abdel‐Tawab, Werz, & Schubert‐Zsilavecz, 2011). The pentacyclic triterpenoids from B. serrata comprising a β‐carboxyl moiety at C‐24 are regarded as biologically/pharmacologically active compounds (Sailer et al., 1996).

The β‐configured pentacyclic triterpene acids in B. serrata include 3‐acetyl‐11‐keto‐β‐boswellic acid (AKBBA), 11‐keto‐β‐boswellic acid (KBBA), β‐boswellic acid (BBA), and 3‐acetyl‐β‐boswellic acid (ABBA), representing major ingredients reaching in the neighborhood of 14% (w/w) in the lipophilic fractions of B. serrata extract (BSE; Büchele, Zugmaier, & Simmet, 2003). Various pharmacological studies indicate that the β‐configured derivatives of boswellic acids (BAs) from BSE possess essentially superior efficacy over the respective α‐isomers (Poeckel & Werz, 2006). Anti‐inflammatory effects of BSE inhibiting carrageenan‐induced paw edemas in animal models are well documented (Siemoneit et al., 2011). Earlier studies also have reported that BSE attenuates inflammatory mediators and oxidative stress and has been used for the treatment of age‐related inflammatory disorders (Umar et al., 2014).

A limited number of clinical studies were performed related to the efficacy and safety of BSE for OA and joint function, particularly for OA of the knee. These studies include several proprietary preparations from BSEs, with different diagnostic measurements, durations, controls, and blindness (Gupta, Samarakoon, Chandola, & Ravishankar, 2011; Kimmatkar, Thawani, Hingorani, & Khiyani, 2003; Sander, Herborn, & Rau, 1998; Sengupta et al., 2008; Sontakke et al., 2007). Although all the above clinical studies have shown beneficial effects for OA of the knee, small number of patients enrolled, shorter study period employed in these studies, absence of placebo control in some studies, and lack of proper characterization of the extract did not meet all rigorous clinical trial criteria to draw definitive conclusions on the usage of BSE for the treatment of knee OA.

In an earlier clinical study, the efficacy of boswellic acid‐containing product (*Boswellin*®) in combination with Curcumin C3 Complex® and ginger extract was demonstrated in the management of OA (Natarajan & Majeed, 2012). No adverse events were recorded. The results of this study clearly indicated that *Boswellin*® is potent for the management of OA in combination with curcuminoids and ginger.

The present study was planned to evaluate the safety and efficacy of *Boswellin*®, a standardized oral supplementation of BSE containing 30% AKBBA and along with three other bioactive β‐boswellic acids, namely, BBA, KBBA, and ABBA, the highly bioactive and pharmacologically relevant components, in newly diagnosed or untreated patients with OA of the knee. This is the first study with BSE conducted for a period of 120 days longer than any other previous clinical trials. In addition to the measurement of standard parameters, radiography was also employed for the assessment of efficacy. Recent studies have shown that circulating concentrations of high‐sensitive C‐reactive protein (hs‐CRP) levels are associated with inflammation and OA progression (Pearle et al., 2007). Other studies also have shown that degeneration of joints was higher among patients who had higher serum CRP (Bonnet & Walsh, 2005). Therefore, in the present study, serum hs‐CRP level was examined to determine the mechanistic insights on the interactions of β‐boswellic acids against the progression of OA of the knee.

## 2 MATERIALS AND METHODS

### 2.1 Ethics and informed consent

This clinical trial was conducted in accordance with the clinical research guidelines established by the Drugs and Cosmetics Act, 1940, of India; Drugs and Cosmetics Rules, 1945, of India; and the International Conference on Harmonization recommended harmonized tripartite guideline regarding Good Clinical Practice. Ethical guidelines were followed for Biomedical Research on Human Participants, 2006, of Indian Council of Medical Research in India and the principles enunciated in the World Medical Association Declaration of Helsinki ethical principles for medical research involving human subjects and adopted by the 18th WMA General Assembly, Helsinki, Finland, June 1964, with the latest amendment in 2004. The study was approved by the institutional ethics committee, written informed consents were obtained from all participants, and the study was registered at a public Clinical Trial Registry in India (www.ctri.nic.in; CTRI/2014/03/004498). No amendments were made to the approved protocol.

### 2.2 Patient recruitment

Both male and female patients, between 35 and 75 years of age, newly diagnosed for OA were screened based on typical history, clinical presentation, classical radiological findings, and fulfilling the classification for OA of the knees according to the criteria of the American College of Rheumatology.

All participants who met the following inclusion criteria were selected for enrolment: (a) patients with a minimum pain visual analog scale (VAS) score >4 on walking in one or both knees during the 24 hr preceding recruitment; (b) patient ambulant and requiring treatment with an anti‐inflammatory drug and not receiving regular anti‐inflammatory or analgesic drugs or not satisfied with drugs being taken and seek a change; (c) patients willing to come for regular follow‐up visits; and (d) participants had to be able to walk and give both verbal and written information regarding the study. Demographic data, physical examination, medical and medication history, comorbid conditions, and vital signs were recorded. All participants provided written informed consent.

The exclusion criteria for the study included the following: (a) known hypersensitivity to herbal extracts or dietary supplements; (b) pregnant or lactating women and women of child bearing potential not following adequate contraceptive measure or women who were found positive for urine pregnancy test; (c) nondegenerative joint diseases or other joint degenerative diseases; (d) incapacitated or bound to wheel chair or bed and unable to carry out self‐care activities; (e) current or recent (in the last 3 months) oral or intra‐articular corticosteroid therapy; (f) preexisting or recent onset of demyelinating disorders or type I diabetes; (g) ongoing with anticoagulants, hydantoin, lithium, steroids, methotrexate, and colchicine; (h) renal, hepatic, or hematopoietic disease or hypertension or severe cardiac insufficiency or congestive heart failure or untreated hyperlipidemia (cardiovascular risk); (i) Ayurvedic formulation or any form of complementary alternative medicine therapy in the preceding 2 months; (j) receiving any investigational drug or participated in any other clinical trial that ended in preceding month or currently ongoing; (k) patients who needed high dose of NSAIDs or analgesics; and (l) inability to comply to study procedures.

### 2.3 Selection of BSE dosage

Information on clinical studies conducted over the past three decades (PubMed database review up to September 2018) was evaluated pertinent to the safety and efficacy of oral administration of BSE in patients with OA or OA of the knee, including rheumatoid arthritis, to select the dosage. In earlier studies, *Boswellia* was given as an extract standardized to contain 30–40% boswellic acids, 300–500 mg two or three times a day (Maroon, Bost, & Maroon, 2010). In a 12‐week pilot study, the authors used (Sander et al., 1998) tablets containing 400 mg of BSE, nine capsules a day (3,600 mg/day) for treatment in outpatients with active rheumatoid arthritis. In a 56‐day crossover study, 333‐mg capsules of BSE containing 40% BAs (corresponding to 118.4 mg of total β‐boswellic acids) were given three times a day (355.2 mg of total β‐boswellic acids per day in addition to α‐boswellic acids) for the treatment of OA of the knee (Kimmatkar et al., 2003). Similarly, 333‐mg capsules of BSE three times a day were also given in a 180‐day trial to compare the efficacy of BSE with valdecoxib (a selective COX‐2 inhibitor) in patients with OA of the knee (Sontakke et al., 2007). Recently, in a 90‐day trial, Sengupta et al. (2008) evaluated the efficacy and safety of BSE (250 mg) enriched with 30% AKBBA (corresponding to 75 mg of AKBBA); however, details of other β‐boswellic acids in the composition were not provided in the treatment of OA of the knee. In a more recent study, patients were administered with 500‐mg capsule of B. serrata, 6 g/day (in three divided doses) of undetermined composition of β‐boswellic acids in the management of OA (Gupta et al., 2011).

In this study, BSE tablets, each tablet containing the BSE extract of 169.33 mg with a mean value of 87.3 mg of total β‐boswellic acids, corresponding to the four major β‐boswellic acids, namely, AKBBA (53.27 mg), BBA (20.83 mg), KBBA (7.11 mg), and ABBA (6.06 mg), were given twice a day. Thus, the selected dosage of BSE, equivalent to 87.3 mg of total β‐boswellic acids per tablet twice a day (174.6 mg of total β‐boswellic acids per day), was safe and was comparable or well below the amount of total β‐boswellic acids in BSE used in previous clinical trials in patients with OA or OA of the knee. The individual boswellic acids in the extract contents were AKBBA ≥ 30%, KBBA ≥ 1.5%, ABBA ≥ 3.5%, and BBA ≥ 7.5% with not less than 50% w/w of total boswellic acids in the extract.

### 2.4 Study design

**Figure 1:**
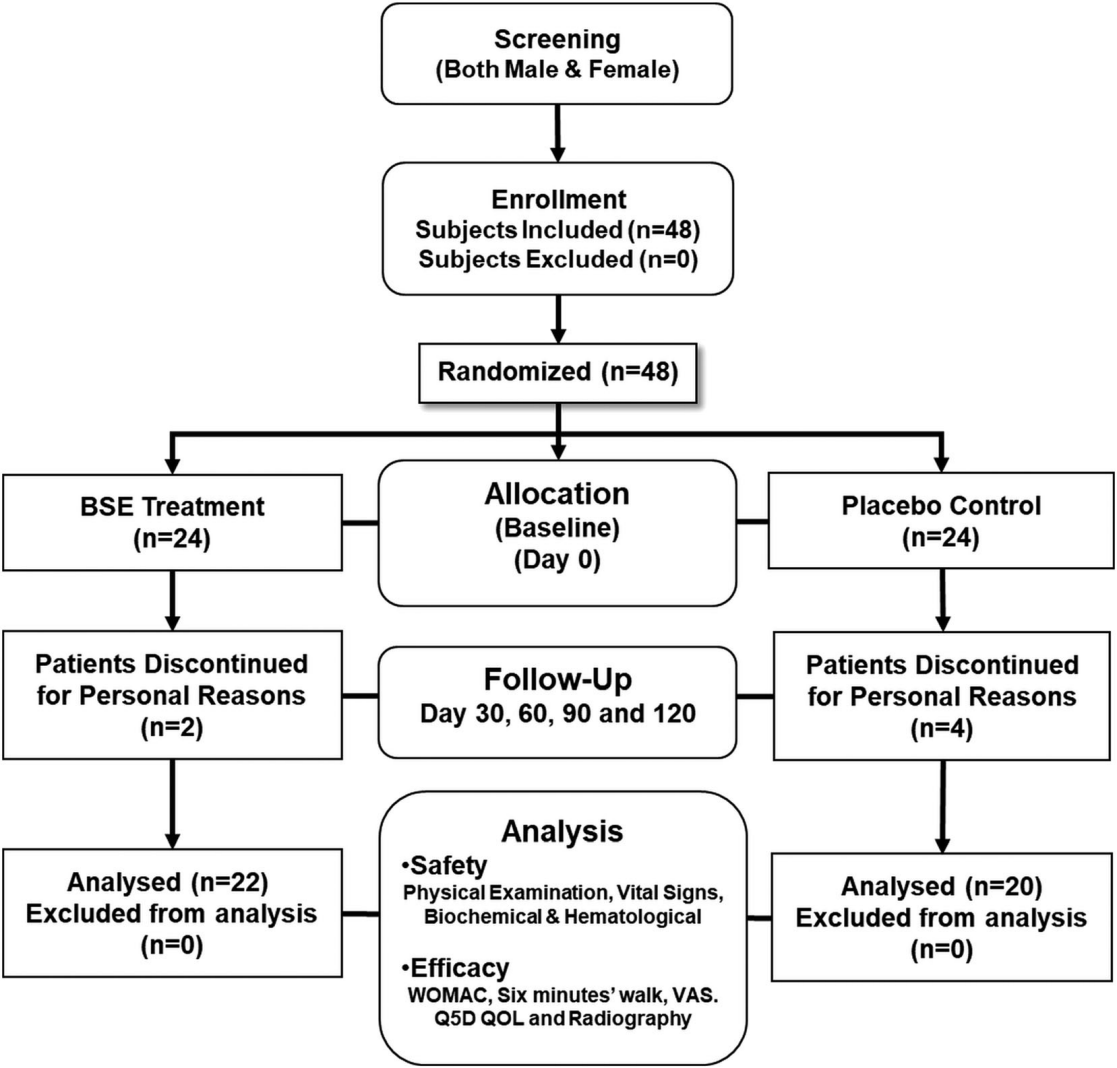
Study design flowchart of Boswellia serrata extract (BSE). A tablet form of BSE (169.33 mg containing 30% 3‐acetyl‐11‐keto‐β‐boswellic acid [AKBBA]) was given orally twice daily for a period of 120 days in patients with osteoarthritis (OA) of the knee

This clinical trial to evaluate the safety and efficacy of the tablet form of BSE in patients with knee OA was performed at the Kempegowda Institute of Medical Sciences, Bangalore, India. Recruitment of patients for this trial commenced on March 18, 2014, and completed on June 6, 2014. A total of 48 newly diagnosed or untreated patients with OA of the knee, with mild to moderate in severity and who were not on any other treatment in the past 3 months, were randomly assigned, in a 1:1 ratio, to receive either BSE or placebo, respectively. Subjects were instructed to self‐administer two tablets of 169.33 mg of BSE each day, each tablet containing a mean value of 87.3 mg of total β‐boswellic acids, or placebo for a period of 120 days (Figure 1). No concomitant medications were allowed.

### 2.5 Randomization and blinding

Both BSE and placebo were coated tablets and were identical to allow for blinding. The coating materials used for both the tablets were exactly the same such that color, taste, and smell are uniform in nature and were packed identically in the same type of bottles. One bottle of tablets was dispensed at each study visit for twice‐daily dosing for 1 month, providing sufficient extra pills to allow visit windows of up to 40 days. During the double‐blinded treatment phase of the study, the subject and all personnel involved with the conduct of the interpretation of the study, including the investigators and investigational site personnel, were blinded to the medication codes. An authorized statistician, independent of the sponsoring organization, not involved in conduct or reporting of the study made random allocation cards using computer‐generated random numbers. The randomization codes were recorded to avoid further confusion, and data were kept strictly confidential. The original random allocation sequences were accessible only to authorized persons on an emergency basis as per sponsor's standard operating procedures until the time of unblinding. Through unblinding of randomization codes at the end of statistical analysis, it was revealed that the XAXA01 group received active BSE (*Boswellin*®) whereas the XAXA02 group received the placebo.

### 2.6 Intervention and compliance

All subjects were asked to take two tablets (either BSE or placebo) per day. Subjects were provided with study visit plan. Study personnel conducted regular home visits to ensure compliance as per the protocol with special reference to medications and follow‐up visits. A diary was provided to the patients to record their daily study and nonstudy medications and any adverse health event. The trial coordinator checked the diary and further ensured compliance. Unused tablets were returned and were analyzed for percent treatment compliance. Out of a total of 48 subjects enrolled into the study, 42 (22 in the BSE group and 20 in the placebo group) completed the study. Six subjects (two from the BSE group and four from the placebo group) dropped out of study, citing personal reasons, and withdrawals from the study, unrelated to treatment effects, were significantly lower for the BSE treatment group than the placebo group (Figure 1).

### 2.7 Outcome measures

The severity of OA based on a QOL questionnaire, radiography, and physical examination before and after BSE treatment was assessed for the efficacy. The Western Ontario McMaster Index (WOMAC) was used for the assessment of pain, stiffness, and physical function in patients with OA of the knee to evaluate the efficacy of at Days 0, 30, 60, 90, and 120. The WOMAC questionnaire contains questions related to severity and frequency of symptoms such as swelling of the joint, grinding and clicking noises, knee catching or hanging up, the ability to straighten or bend knees, pain in the knees in different positions, knee functions, and the ability to perform daily functions. Based on the sum of all the scores, the overall WOMAC score was determined.

The objective of the 6‐min walk test was to evaluate the effect of BSE on the ability of the patients to walk as far as possible for 6 min. The distance travelled by patients/study subjects in a time period of 6 min was recorded on the baseline and during other study visits. Other measures performed included determination of physician's and subject's global assessment, 6‐min walk test, VAS pain scores, and European Quality of life‐5 Dimension QOL. The physical exam was focused on the range of motion (both passive and active), muscle strength, ligament stability, and tenderness of the affected joints. A comparative analysis of radiological X‐ray images captured before (baseline) and on Day 120 was performed to determine the efficacy of BSE treatment. All radiographs were obtained under standardized conditions.

For the analysis of serum hs‐CRP, blood samples were collected from subjects on scheduled visits. Fresh serum sample was prepared by centrifugation after 1‐hr interval at room temperature. hs‐CRP was measured by a particle‐enhanced immunoturbidimetric assay using commercially available kit in which human CRP agglutinates with latex particles coated with monoclonal anti‐CRP antibodies. The precipitate was determined turbidimetrically on a Roche/Hitachi cobas c 501/502 using reagents/kit from Roche Diagnostics GmbH (Mannheim, Germany). The lower detection limit of the hs‐CRP assay was 0.03 mg/L, and measurements lower than 0.03 mg/L were not considered.

### 2.8 Safety assessment

Vital signs, namely, blood pressure, respiratory rate, pulse rate, and any abnormal lab/diagnostic parameters, were considered for safety evaluations. Physical examination and vital signs were measured on Days 0, 30, 60, 90, and 120. Demographic data were recorded on Days 0 and 120. Vital signs were assessed immediately after BSE tablets were taken for the first time and continued throughout the study. The routine laboratory parameters of safety, namely hematological and biochemical investigations, were measured using standard laboratory techniques, before and after the BSE treatment. Urine test for pregnancy was performed on female volunteers of child bearing potential. Adverse effects, if any, were recorded at each study visit.

### 2.9 Statistical analysis

All data are expressed as mean ± *SD*. Data were evaluated for statistical significance by *t* test or analysis of covariance or Wilcoxon's signed rank sum test depending on the number of comparisons made to reach the best possible statistical conclusion between patients receiving BSE and placebo. Last observation carried forward method was followed for efficacy evaluations of subjects, whose data were not available in the last/final visit. Results with *p <* 0.05 are considered statistically significant. Statistical Analysis Software (SAS) of version 9.2 (Cary, NC, USA) was used for data analysis.

## 3 RESULTS

### 3.1 Demographic characteristics of subjects

Details on the overall demographic characteristics of subjects enrolled for the trial are provided in Table 1. A comparative detail on the body mass index, height, and weight of subjects recorded at the baseline and Day 120 of BSE treatment is presented in Table 2. No significant differences were observed in the demographic characteristics between the group who received 169.33 mg of tablets of BSE and the placebo.

**Table 1:** Demographic characteristics of subjects selected for the *Boswellia serrata* extract intervention trial

Parameter	Statistics	Total no. of subjects (*N* = 48)
Age (years)	Mean ± *SD*	58.7 ± 8.20
Height (cm)	Mean ± *SD*	165.3 ± 9.82
Weight (kg)	Mean ± *SD*	63.9 ± 11.12
Body mass index (kg/m^2^)	Mean ± *SD*	23.31 ± 4.13
Gender		
Male	*N* (%)	17 ± 35.42
Female	*N* (%)	31 ± 64.58

*Note*. Values are presented as mean ± *SD*.

**Table 2:** BMI, height, and weight of subjects recorded between the baseline and after BSE treatment

	BSE			Placebo			*p*‐value BSE versus placebo
Parameter	Baseline (*N* = 24)	Day 120 (*N* = 22)	*p* value	Baseline (*N* = 24)	Day 120 (*N* = 20)	*p* value	
BMI (kg/m^2^; mean ± *SD*)	23.37 ± 3.95	21.0 ± 3.95	0.7574	23.25 ± 4.48	23.4 ± 3.68	0.6967	0.1478
Height (cm; mean ± *SD*)	165.2 ± 12.54	166.2 ± 13.24	0.6801	165.5 ± 6.60	163.6 ± 6.28	0.8797	0.4181
Weight (kg; mean ± *SD*)	64 ± 12.40	67.1 ± 12.19	0.8464	63.9 ± 9.00	63.3 ± 0.01	0.7644	0.4290

*Note*. Values are presented as mean ± *SD*. One‐way analysis of variance test was performed between the baseline and after treatment (Day 120) of each group and between the BSE treatment and placebo groups. *p* values are not significant (*p* > 0.05). BMI: body mass index; BSE: *Boswellia serrata* extract.

### 3.2 Clinical efficacy

**Figure 2:**
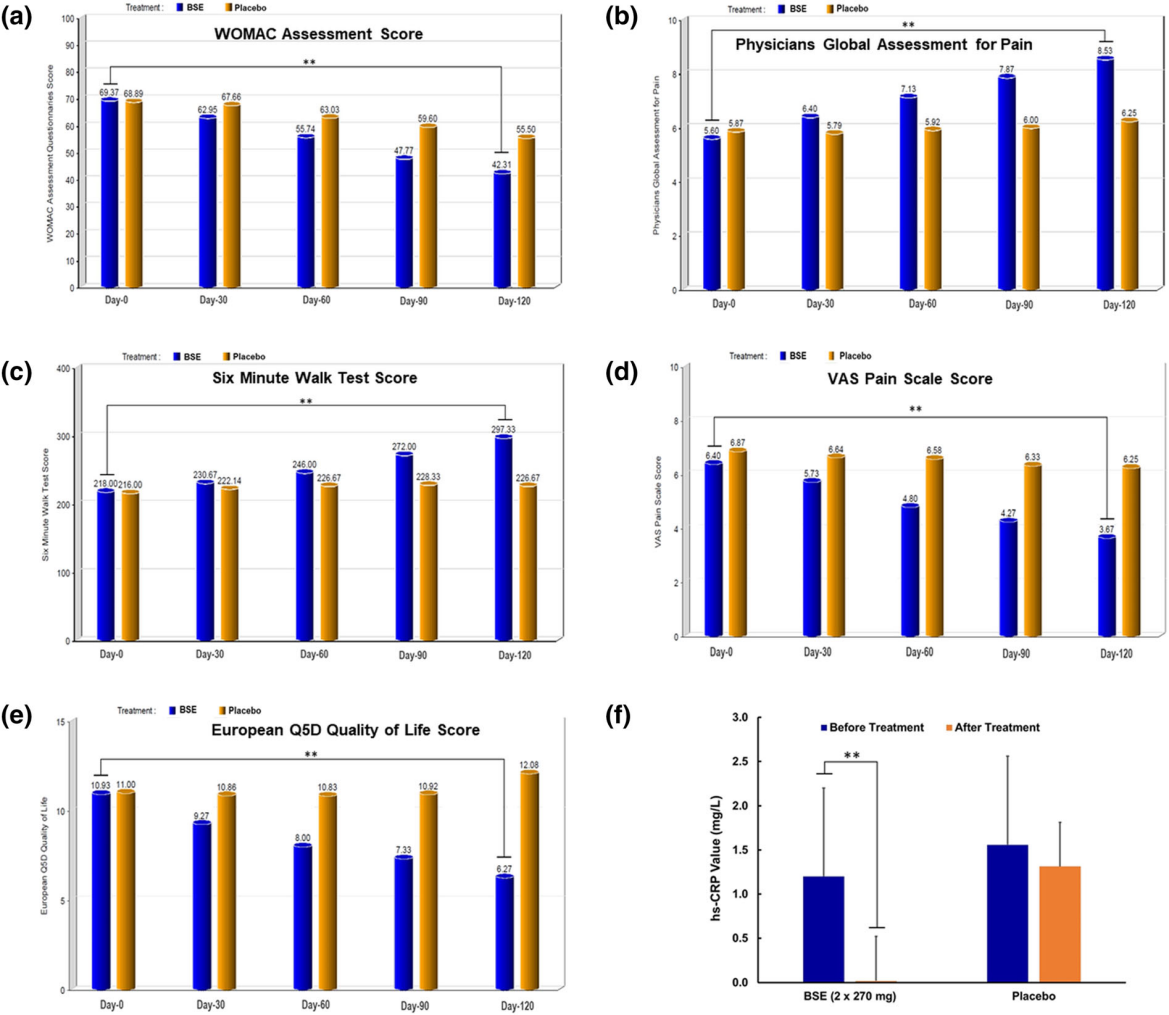
Bar graphs show the efficacy analyses with Boswellia serrata extract (BSE) treatment. (a–e) The efficacy bar graphs are based on quality of life (QOL) questionnaire scores in patients with osteoarthritis (OA) of the knee treated with BSE (Day 120) compared between placebo control and their baseline visit (Day 0). (a) BSE treatment shows a significant decrease in Western Ontario McMaster Index (WOMAC) score indicating improvements in pain, stiffness, and physical function; (b) Physician Pain Assessment Scale also shows significant better score with BSE treatment; (c) the trend in patients' walking ability shows improvement with BSE treatment; (d) reduction in the visual analog scale (VAS) pain scale score in the BSE group was conferred by Day 120; (e) European Quality of life‐5 Dimension quality of life score indicated patient's health, good/better QOL, with BSE treatment; (f) bar graph shows the effect BSE on high‐sensitive C‐reactive protein (hs‐CRP), between and within treatment groups on the baseline and Day 120 of the BSE treatment and placebo (control) groups in patients with OA of the knee. ^**^Statistically significant (*p* < 0.01) [Colour figure can be viewed at wileyonlinelibrary.com]

The data on the efficacy assessments, including WOMAC, Physician's Global Assessment, 6‐min walk test, and VAS scores after 120 days of BSE treatment, are presented in Figure 2. Detailed analyses pertinent to the overall WOMAC scores, Physician's Global Assessment, 6‐min walk test, and VAS scores are presented in Table 3. Analysis of covariance was applied to confirm the efficacy assessment. Mean scores were used for bilateral OA. The differences in the efficacy parameters between the baseline and after BSE treatment group compared with the placebo group were found to be significant (*p* < 0.001). A similar difference (*p* < 0.001) was also observed when nonparametric Wilcoxon test was employed. However, no significant difference was observed between the baseline and the placebo group.

**Table 3:** Comparative analysis of 120‐day efficacy measures with BSE versus placebo

Parameter	BSE			Placebo			*p* value BSE versus placebo
	Baseline	Day 120	*p* value	Baseline	Day 120	*p* value	
WOMAC assessment questionnaires score	69.4 ± 8.06	42.3 ± 4.84**	0.0001	68.9 ± 7.48	55.5 ± 6.72	0.0003	0.0001
Physician's Global Assessment for pain	5.6 ± 0.91	8.5 ± 0.64**	0.0001	5.9 ± 1.19	6.3 ± 0.87	0.2175	0.0001
Six‐minute walk test score	218.0 ± 26.51	297.3 ± 27.89**	0.0001	216.0 ± 28.23	226.7 ± 27.41	0.7082	0.0001
VAS pain scale score	6.4 ± 1.24	3.7 ± 1.35**	0.0001	6.9 ± 1.51	6.3 ± 0.62	0.1828	0.0001
European Q5D quality of life	10.9 ± 1.71	6.3 ± 0.88**	0.0001	11.0 ± 1.20	12.1 ± 1.93	0.0858	0.0001

*Note*. Values are presented as mean ± *SD*. One‐way analysis of variance test was performed between the baseline and after treatment (Day 120) of each group and between the BSE treatment and placebo groups. BSE: *Boswellia serrata* extract; VAS: visual analog scale; WOMAC: Western Ontario McMaster Index.

** *p* values are significant (*p* < 0.0001).

### 3.3 Effect of BSE treatment on WOMAC pain score

A comparative analysis of WOMAC score at the baseline visit showed a mean value of 69.4 ± 8.06 and 68.9 ± 7.48 for the treatment and placebo groups, respectively. However, a steady decrease in the WOMAC score was observed at different time points in patients with the BSE treatment, and a mean value of 42.3 ± 4.84 was observed on Day 120. The mean WOMAC score for the placebo group was 55.5 ± 6.72 on Day 120. Overall, the BSE treatment group showed a statistically significant (*p* < 0.001) decrease in WOMAC score indicating improvements in physical function by reducing pain and stiffness (Figure 2a and Table 3). The subscores of WOMAC, namely, the three domains of pain, physical function, and stiffness, are provided in Table S4. The values of the subscores of WOMAC are in agreement with the WOMAC overall score that BSE treatment significantly reduced the pain and stiffness compared with placebo control in patients with OA of the knee.

### 3.4 Effect of BSE treatment on Physician's Global Pain Assessment scale

Pain assessment scale ranging from 0 to 10, where 0 indicates *very poor* and 10 *excellent*, was used by the physician for assessment. At the baseline visit, the mean values were found to be 5.6 ± 0.91 and 5.9 ± 1.19 between the BSE treatment and placebo groups, respectively. At the end of the study, patients in the BSE treatment presented a mean score of 8.5 ± 0.64, which is a statistically significant better score (*p* < 0.001), compared with their baseline visit values, but also significantly different from the mean values of patients in the placebo group (6.3 ± 0.87), on the final visit (Figure 2b and Table 3).

### 3.5 Effect of BSE treatment on the ability to walk

As per this assessment, the distance travelled by subjects in a period of 6 min was recorded. On the baseline visit, a mean of 218.0 ± 26.51 and 216.0 ± 28.23 m was recorded for the BSE treatment and placebo groups, respectively. At the end of the study, the trend in walking had improved significantly (*p* < 0.01) in patients of the BSE treatment group, with a mean value of 297.3 ± 27.89 m, whereas it was 226.7 ± 27.41 m in patients of the placebo group. The difference in the efficacy assessments was significant (*p* < 0.001) between the groups when their respective final visit (Day 120) values were analyzed (Figure 2c and Table 3).

### 3.6 Effect of BSE treatment on VAS pain scale

The VAS pain scale score was significantly reduced after BSE treatment. Briefly, on the baseline visit, a mean score of 6.4 ± 1.24 and 6.9 ± 1.51 was reported by the active and placebo treatment groups of patients, respectively. On Day 120 (final visit), the pain score decreased significantly (*p* < 0.001) to 3.7 ± 1.35 in the active treatment group of patients with no statistically significant change, whereas it was 6.3 ± 0.62 in the placebo receiving patients. The study concluded that as per physicians' assessment, patients felt much better with BSE (active) when compared with placebo (Figure 2d and Table 3).

### 3.7 Effect of BSE treatment on European Quality of life (QOL)‐5 Dimension score measures

This is a self‐report QOL that measures mobility, self‐care, usual activities, pain/discomfort, and anxiety/depression. This questionnaire indicates how bad a patient's health is on a specific day. A total score of 15 indicates poor QOL, whereas 5 indicates good/better QOL. Mean values of 10.9 ± 1.71 and 11.0 ± 1.20 on baseline visits were changed to 6.3 ± 0.88 and 12.1 ± 1.93 on the final visit between the active BSE and placebo receiving groups of patients, respectively (Figure 2e and Table 3). The differences in the QOL between the baseline and after BSE treatment group compared with the placebo group were found to be significant (*p* < 0.001).

### 3.8 Effect of BSE treatment on radiological X‐ray examination

**Figure 3:**
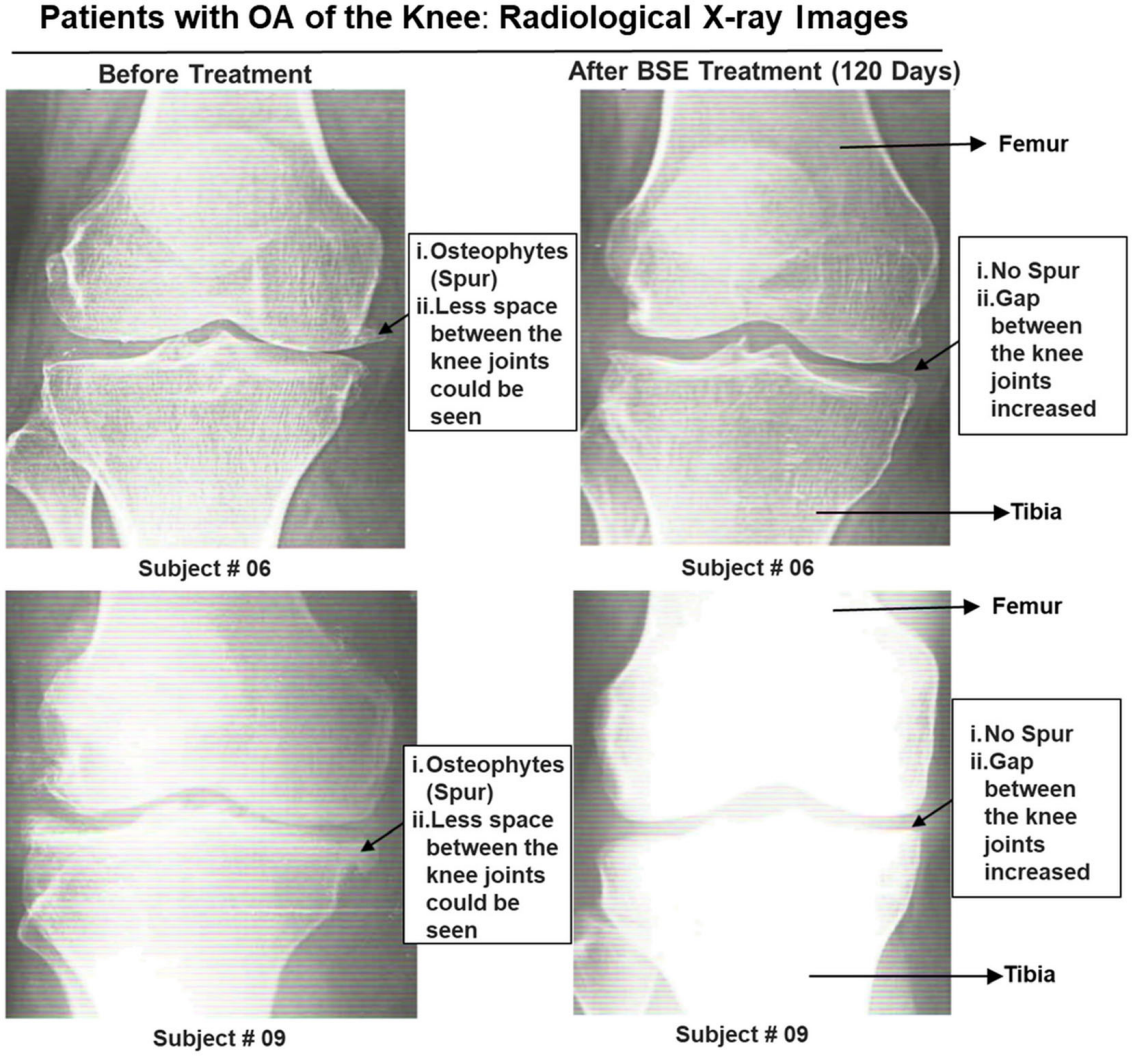
Radiological X‐ray images of the knee of patients (# 06 and 09) captured before and after Boswellia serrata extract (BSE) treatment. Significant improvements in patients with osteoarthritis (OA) of the knee conditions were observed, where the gap between the knee joints increased significantly with decrease in osteophytes (spur) with BSE treatment [Colour figure can be viewed at wileyonlinelibrary.com]

Examination of radiological X‐ray images revealed reduced joint space due to loss of articular cartilage and osteophyte formation in OA of the knee patients in the placebo group. On the other hand, the OA of the knee patients in the BSE treatment groups showed significant improvements on the final visit (120 days). A distinct change in the OA condition could be seen where the gap between the knee joints increased significantly with a sharp decrease in osteophytes (spur) in subjects (Figure 3).

### 3.9 Effect of BSE treatment on hs‐CRP

Elevated levels of hs‐CRP are found to be associated with local inflammation in patients with OA. Several studies have shown that hs‐CRP is elevated in the plasma of patients with OA compared with the age‐matched controls (Pearle et al., 2007). In this study, a significant decrease in the activity of hs‐CRP in the BSE‐treated group was observed in contrast to that from the placebo receiving group (*p* < 0.01; Figure 2f). These findings are clearly suggesting that BSE is a powerful inhibitor of hs‐CRP induced by local inflammation in patients with OA.

### 3.10 Safety evaluations

None of the enrolled subjects had abnormal medical history. No abnormality in physical findings was observed on the screening visit or during the study visits. The vital signs recorded in listings for physical examination did not show statistically significant changes that are recorded between the baseline and Day 120 of BSE or between the treatment groups (Table S1). Systolic blood pressure and diastolic blood pressure pulse rate, respiratory rate, heart rate, and oral temperature were normal on the screening visits and during the study visits as well.

### 3.11 Biochemical and hematological evaluations

As part of the safety evaluation, a complete set of analysis was performed for (a) the biochemical parameters in the urine and in the serum samples and (b) for hematological parameters (before and at the end of the study). These analyses are summarized in Tables S2 and S3. A repeated measure analysis of variance test was performed to compare the values of the above parameters at recorded at different time points of the visit (baseline and at the final visit). Statistical analysis of the data for the biochemical and hematological parameters did not indicate any significant changes. Some of the minor changes (if any) observed were found to be within the normal laboratory range.

### 3.12 Adverse events

There were no statistically significant changes in the body weight and body mass index from baseline to the last visit or between the treatment groups. Vital signs, namely, blood pressure, respiratory rate, pulse rate, and any abnormal lab/diagnostic parameters recorded, were safe and thus support the safety of the agent BSE. During the course of the study, there were no serious adverse events reported. No clinically significant abnormal lab values were identified, and no statistically significant changes in the vitals were observed from the baseline to the final visits. Although few high values were reported, they were categorized as “not clinically significant” by the study investigator owing to their marginal borderline values from the lab reference ranges. The percentage of treatment compliance for 42 patients who completed the study was good. The events were resolved and closed, and the subjects completed all remaining study visits. No concomitant medications were allowed.

## 4 DISCUSSION

The present clinical trial demonstrated the safety and efficacy of oral supplementation of BSE containing 30% AKBBA and other bioactive β‐boswellic acids, namely, BBA, KBBA, and ABBA, in newly diagnosed or untreated patients with OA of the knee. Use of herbal medicines and phytonutrients or nutraceuticals is gaining interest in therapies, including prevention and treatment of chronic diseases such as OA. In this context, Panahi et al. (2014) have shown that curcuminoids significantly reduce WOMAC, VAS, and Lequesne's pain functional index scores and concluded that curcuminoids also represent an effective and safe alternative treatment for OA. Earlier studies using knee cartilage at in vitro conditions demonstrated the anti‐arthritic effects of an Ayurvedic formulation containing Zingiber officinale root, *Tinospora cordifolia* stem, Phyllanthus emblica fruit, and oleoresin of B. serrata (Sumantran et al., 2011). Specifically, oleoresin of B. serrata was shown to play a crucial role in the chondroprotective and anti‐inflammatory activity in OA patients (Sumantran et al., 2011). An earlier study using a triterpene‐rich extract of Vitellaria paradoxa against OA showed a decrease in tumor necrosis factor alpha and a cartilage degradation marker CTX‐II (Cheras, Myers, Paul‐Brent, Outerbridge, & Nielsen, 2010). Likewise, a clinical study on the efficacy of green tea extract conducted in patients with OA of the knee for 4 weeks showed a reduction in VAS pain, total WOMAC, and WOMAC physical function scores compared with the control group. However, the authors reported no significant differences between the two groups and suggested that future studies with longer duration in larger sample size may be required to validate the efficacy (Hashempur, Sadrneshin, Mosavat, & Ashraf, 2018). Despite the existence of various plant products, sustainable use of plant resources in the management of OA is still challenging.

Earlier, the efficacy of boswellic acid‐containing product (*Boswellin*®) in combination with Curcumin C3 Complex® and ginger extract was demonstrated in the management of OA (Natarajan & Majeed, 2012). No adverse events were recorded. The present study demonstrated that oral supplementation of BSE containing AKBBA and BBA significantly improved physical function by reducing pain and stiffness compared with placebo control in newly diagnosed or untreated patients with OA of the knee, as presented in Table 3 and Figure 2. Also, radiographic assessment showed that BSE significantly improved between the knee joints and reduced osteophytes (spur) formation compared with the placebo (Figure 3), thus, confirming the efficacy of BSE against OA of the knee (Figure 3). More importantly, BSE treatment comprising 30% AKBBA and BBA significantly decreased hs‐CRP values compared with the placebo group (Figure 2f) clearly supporting the clinical efficacy for OA.

**Figure 4:**
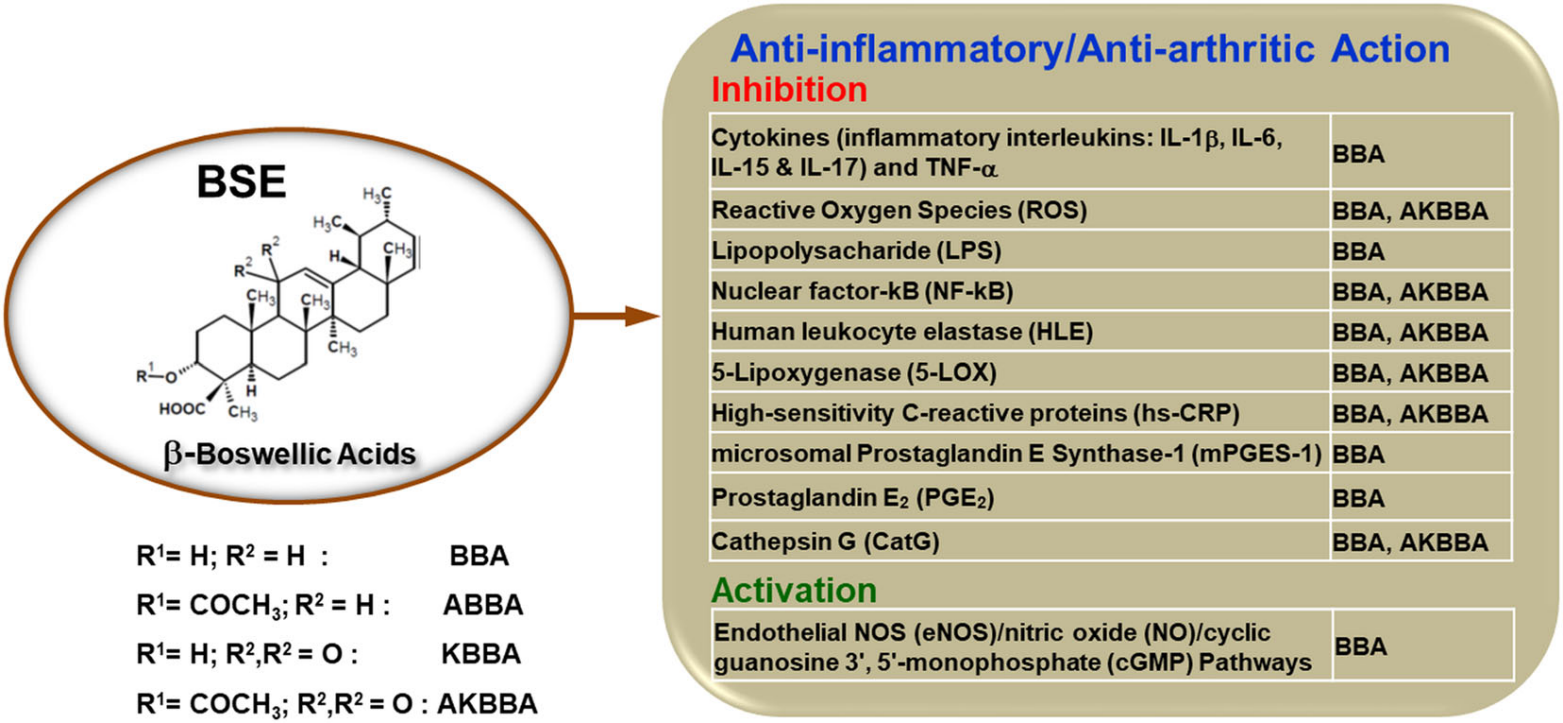
Mechanisms of action of β‐boswellic acids against osteoarthritis (OA) of the knee. Biologically active constituents of BSE, namely, β‐boswellic acid (BBA) and 3‐acetyl‐11‐keto‐β‐boswellic acid (AKBBA), act synergistically to exert anti‐inflammatory/anti‐arthritic activity to reduce joint pain and improve physical functional ability in patients with OA of the knee. ABBA: 3‐acetyl‐β‐boswellic acid; IL: interleukin; KBBA: 11‐keto‐β‐boswellic acid; TNF‐α: tumor necrosis factor alpha [Colour figure can be viewed at wileyonlinelibrary.com]

Regarding structure and functional aspects of BSE, earlier reports suggest that BBA lacking keto functional groups may reverse or partially prevent the activity of AKBBA on 5‐lipoxygenase (5‐LOX) pathway (Safayhi, Sailer, & Ammon, 1995; Sailer et al., 1996). Although the importance of 5‐LOX inhibition by AKBBA in BSE is decidedly important, the findings of the present study provide strong clinical evidence to support the fact that the active components of BSE, namely, BBA and AKBBA, acted synergistically to exert anti‐inflammatory activity efficaciously in reducing joint pain and improving the physical functional ability in patients with knee OA. The current findings are also consistent with earlier studies indicating that BSE comprising β‐configured derivatives of boswellic acids are specific nonredox inhibitors of 5‐LOX and hence inhibit leukotriene biosynthesis and reduce the pain associated with joint stiffness and physical discomfort (Gupta et al., 2011; Kimmatkar et al., 2003; Sengupta et al., 2008; Sontakke et al., 2007) (Figure 4). Although human leukocyte elastase activities inhibition is established for many lipophilic compounds, a dual human leukocyte elastase and 5‐LOX inhibitory property is unique to pentacyclic triterpenes (Safayhi, Rall, Sailer, & Ammon, 1997). Although AKBBA has been reported as a natural inhibitor of the transcription factor nuclear factor κB involved in inflammatory reactions (Cuaz‐Pérolin et al., 2008), BSE has been shown to reduce the production of reactive oxygen species in OA‐related oxidative stress conditions (Umar et al., 2014).

Although AKBBA and KBA have been considered as the pharmacologically active ingredients (Safayhi et al., 1992), recent studies show that β‐boswellic acids, including BBA lacking the C11‐oxo moiety, are about equipotent to 11‐keto‐β‐boswellic acids to interfere with serine protease cathepsin G (Tausch et al., 2009). The potent interference of BBA with cathepsin G in relation to its higher achievable plasma levels favors this interaction as a possible molecular basis for the underlying beneficial effects of BSE (Figure 4).

Although NSAIDs can cause disruption of glycosaminoglycan synthesis, BSE has been claimed to decrease the glycosaminoglycan degradation (Reddy, Chandrakasan, & Dhar, 1989), which in turn may help to keep the cartilage in good condition and stop the progression of OA of the knee. Another possible mode of action of BSE could be inhibiting microsomal prostaglandin E synthase‐1 induced by proinflammatory stimuli such as interleukin 1b or lipopolysaccharide (LPS), especially by BBA (Siemoneit et al., 2011) (Figure 4).

It is worth mentioning that BBA significantly increases nitric oxide and cyclic guanosine 3′,5′‐monophosphate levels in carotid aortas of blood stasis rats. The protective effect of BBA via endothelial nitric oxide synthase signaling pathway was demonstrated on human umbilical vein endothelial cells exposed to transient oxygen and glucose deprivation. Thus, BBA could counteract the effect on platelet aggregation (Wang et al., 2015). Additionally, pretreatment of BBA results in a significant decrease of blood endothelin‐1 level, a key player in endothelial dysfunction, which further provides a better insight into the BBA's protective mechanism on endothelial functions. Among the naturally occurring BAs in BSE, BBA might be the most relevant BA derivative that inhibits LPS‐induced responses (Pandey, Singh, & Tripathi, 2005; Syrovets, Büchele, Krauss, Laumonnier, & Simmet, 2005), because the concentration of BBA required for efficient inhibition of LPS activity is clearly in the range of blood plasma levels reached (10.1 μM) after oral application of standard doses of BSE (Büchele et al., 2003; Tausch et al., 2009). Based on blood plasma levels of BBA, BSE has been shown to be effective in diseases connected to elevated LPS levels, and neutralization of LPS by BBA may be a part of the molecular actions responsible for the beneficial BSE effects (Henkel et al., 2012) (Figure 4).

Importantly, findings of the current study show that BSE significantly reduced the serum levels of hs‐CRP providing new insights on the potential anti‐inflammatory mode of action of BSE (Figure 4). Regarding clinical safety, assessment of the laboratory/diagnostic parameters observed in this study confirms that there were no serious adverse events with BSE treatment.

The current study has a few limitations. This is a pilot study with a small group of subjects. However, studies with larger human cohort is required to confirm the conclusions of the present study. hs‐CRP, a potential inflammatory marker associated with OA of the knee, was used to assess the anti‐inflammatory activity of BSE. Use of a panel of inflammatory markers, including matrix metalloproteinase‐derived inflammation, a component of OA (Siebuhr et al., 2014), and interleukin 6, identified in the systemic circulation and synovial fluid of OA patients (Bonnet & Walsh, 2005), may provide distinctive effects of BSE on inflammation associated with OA. Despite some of these limitations, the present clinical trial demonstrated the safety and efficacy of BSE (*Boswellin*®), in patients with OA of the knee.

## 5 CONCLUSIONS

The findings from the present study provide clinical evidence to support that biologically active components of BSE, specifically AKBBA and BBA, acted synergistically to exert anti‐inflammatory/anti‐arthritic activity efficaciously in reducing joint pain and improving the physical functional ability (Figure 4). No serious adverse events were observed, thus supporting the pharmacological safety of BSE (*Boswellin*®) to be considered as a viable candidate for the treatment of OA of the knee.

## CONFLICT OF INTEREST

All the authors are affiliated to Sami Labs Limited or Sabinsa Corporation. Sabinsa Corporation and Sami Labs hold patents/trademarks covering *Boswellin*®.

## Supporting information


**Table S1:** Assessment of 120‐day vital safety parameters with BSE vs. Placebo
**Table S2**: Assessment of 120‐day Biochemical safety parameters with BSE treatment vs. Placebo
**Table S3**: Assessment of 120‐day hematological safety parameters with BSE treatment vs. Placebo
**Table S4:** Comparative analysis of sub‐scores of WOMAC, baseline vs. 120‐day, for efficacy measures with BSE treatment.
