# Supplementary material for: A pilot, randomized, double‐blind, placebo‐controlled trial to assess the safety and efficacy of a novel Boswellia serrata extract in the management of osteoarthritis of the knee
Source: Phytother Res. 2019 Mar 6;33(5):1457–68. doi: 10.1002/ptr.6338 (PMC6681146; doi:10.1002/ptr.6338)
Supplement: Supplementary file 1 — Table S1: Assessment of 120‐day vital safety parameters with BSE vs. Placebo Table S2: Assessment of 120‐day Biochemical safety parameters with BSE treatment vs. Placebo Table S3: Assessment of 120‐day hematological safety parameters with BSE treatment vs. Placebo Table S4: Comparative analysis of sub‐scores of WOMAC, baseline vs. 120‐day, for efficacy measures with BSE treatment. [file PTR-33-1457-s001.docx]

**Majeed et al.**

**Supplementary Table 1:** Assessment of 120-day vital safety parameters with BSE vs. Placebo

| **Vital Parameter** | **Treatment** | **Baseline**  **(Day 0)** | **Visit 5**  **Day 120)** | **p-value** |
| --- | --- | --- | --- | --- |
| Systolic Blood Pressure (mmHg) | BSE | 134.4 | 136.9 | 0.2675 |
|  | Placebo | 130.7 | 134.5 | 0.0123 |
| Diastolic Blood Pressure (mmHg) | BSE | 86.1 | 82.8 | 0.041 |
|  | Placebo | 82.6 | 82.3 | 0.7357 |
| Heart Rate (Beats per minute) | BSE | 74.1 | 75.5 | 0.1053 |
|  | Placebo | 74.9 | 75.6 | 0.4124 |
| Pulse Rate (Beats per minute) | BSE | 74.1 | 75.5 | 0.0835 |
|  | Placebo | 75.2 | 74.8 | 0.8153 |
| Respiratory Rate (Breaths per minute) | BSE | 19.8 | 21.8 | 0.036 |
|  | Placebo | 19.3 | 20 | 0.5884 |
| Oral Temperature (degrees Fahrenheit) | BSE | 98.1 | 98.4 | 0.1021 |
|  | Placebo | 98.1 | 98.5 | 0.0072 |

Data presented as mean values

No significant changes in the vital signs were observed

**Majeed et al.**

**Supplementary Table 2**: Assessment of 120-day Biochemical safety parameters with BSE treatment vs. Placebo

|  | **BSE** | | |  | **Placebo** | | |
| --- | --- | --- | --- | --- | --- | --- | --- |
| **PARAMETER** | **Visit 1 (Day 0) (Mean ± SD)** | **Visit 5 (Day 120) (Mean ± SD)** | **p-value** |  | **Visit 1 (Day 0) (Mean ± SD)** | **Visit 5 (Day 120) (Mean ± SD)** | **p-value** |
| Serum Creatinine (mg/dl) | 0.769 ± 0.144 | 0.815 ± 0.141 | 0.387 |  | 0.863 ± 0.201 | 0.763 ± 0.174 | 0.361 |
| Aspartate aminotransferase (IU/L) | 18.769 ± 8.146 | 20.231 ± 7.507 | 0.318 |  | 17.818 ± 8.976 | 17.910 ± 7.148 | 0.928 |
| Alanine aminotransferase (IU/L) | 22.692 ± 6.920 | 23.846 ± 6.479 | 0.178 |  | 19.455 ± 6.440 | 20.000 ± 5.235 | 0.479 |
| Alkaline Phosphatase (IU/L) | 76.00 ± 26.115 | 82.385 ± 14.210 | 0.419 |  | 88.182 ± 27.672 | 91.091 ± 26.674 | 0.133 |
| Total Bilirubin (mg/dl) | 0.618 ± 0.472 | 0.488 ± 0.259 | 0.381 |  | 0.373 ± 0.144 | 0.441 ± 0.238 | 0.347 |
| Uric Acid (mg/dl) | 4.677 ± 0.741 | 4.631 ± 0.771 | 0.661 |  | 5.109 ± 1.850 | 5.082 ± 1.800 | 0.676 |
| Sodium (mEq/L) | 138.154 ± 1.994 | 138.692 ± 3.497 | 0.594 |  | 135.818 ± 3.430 | 140.000 ± 2.966 | 0.0007 |
| Potassium (mEq/L) | 7.477 ± 10.993 | 7.431 ± 10.708 | 0.991 |  | 8.364 ± 11.832 | 4.373 ± 0.610 | 0.288 |
| Chloride mEq/L) | 97.846 ± 2.193 | 97.923 ± 3.989 | 0.934 |  | 97.727 ± 3.663 | 99.545 ± 3.671 | 0.244 |

Values presented as mean ± S.D

No significant changes in the biochemical safety parameters were observed

**Majeed et al.**

**Supplementary Table 3**: Assessment of 120-day hematological safety parameters with BSE treatment vs. Placebo

|  | **BSE** | | |  | **Placebo** | | |
| --- | --- | --- | --- | --- | --- | --- | --- |
| **PARAMETER** | **Visit 1 (Day 0) (Mean ± SD)** | **Visit 5 (Day 120) (Mean ± SD)** | **p-value** |  | **Visit 1 (Day 0) (Mean ± SD)** | **Visit 5 (Day 120) (Mean ± SD)** | **p-value** |
| Haemoglobin (gm%) | 13.223 ± 1.655 | 13.808 ± 1.791 | 0.062 |  | 13.318 ± 1.096 | 13.518 ± 0.842 | 0.236 |
| Haematocrit (%) | 40.523 ± 3.707 | 41.469 ± 4.788 | 0.361 |  | 41.427 ± 1.730 | 40.845 ± 1.790 | 0.275 |
| Erythrocyte Count (RBC) (Mil/cum) | 19.355 ± 18.850 | 15.315 ± 13.786 | 0.294 |  | 10.796 ± 7.391 | 11.727 ± 7.114 | 0.597 |
| Platelet Count (Lakh/cum) | 2.681 ± 0.487 | 4.926 ± 8.496 | 0.364 |  | 2.370 ± 0.748 | 2.671 ± 0.871 | 0.118 |
| Luekocyte Count (WBC) (cells/cmm) | 14559.460 ± 22356.020 | 8159.846 ± 1473.503 | 0.318 |  | 8741.818 ± 1740.867 | 8546.727 ± 2085.033 | 0.616 |

Values presented as mean ± S.D

No significant changes in the hematological safety parameters were observed

**Majeed et al.**

**Supplmentary Table 4:** Comparative analysis of sub-scores of WOMAC, baseline vs. 120-day, for efficacy measures with BSE treatment.

| **Parameter** | **BSE** | | |  | **Placebo** | | |
| --- | --- | --- | --- | --- | --- | --- | --- |
|  | **Baseline** | **Day-120** | **p-value** |  | **Baseline** | **Day-120** | **p-value** |
| Pain | 13.23 ± 2.09 | 12.08 ± 1.32** | 0.0210 |  | 13.27 ± 2.10 | 11.09 ± 2.98 | 0.055 |
| Stiffness | 5.46 ± 0.97 | 4.23 ± 0.979** | 0.0430 |  | 5.55 ± 0.93 | 5.18 ± 0,98 | 0.500 |
| Physical Function | 46.00 ± 5.08 | 40.77 ± 4.62** | 0.0002 |  | 45.45 ± 4.89 | 41.00 ± 7.59 | 0.03 |

Values presented as mean ± S.D

**p value significant (<0.01) between the baseline and visit 5 (day-120) in the treatment group.
